# Neoadjuvant anti-tumor vaccination prior to surgery enhances survival

**DOI:** 10.1186/s12967-014-0245-7

**Published:** 2014-09-04

**Authors:** Scott A Fisher, Amanda Cleaver, Devina D Lakhiani, Andrea Khong, Theresa Connor, Ben Wylie, W Joost Lesterhuis, Bruce WS Robinson, Richard A Lake

**Affiliations:** School of Medicine & Pharmacology, University of Western Australia, Perth, 4th Floor, G Block, Queen Elizabeth II Medical Centre, Perth, WA 6009 Australia; National Research Centre for Asbestos Related Diseases, Harry Perkins Institute of Medical Research, QQ Block, 6 Verdun Street, Nedlands, WA 6009 Australia

**Keywords:** Surgery, Cancer vaccine, Lymphocytes, Cancer immunotherapy, Neoadjuvant

## Abstract

**Background:**

This study was conducted to determine if anti-tumor vaccination administered prior to partial debulking surgery could improve survival using a murine solid tumour model.

**Methods:**

Tumor incidence and survival rates were compared in mice bearing subcutaneous AB1-HA mesothelioma tumors that received either sham surgery, debulking surgery or vaccination prior to debulking surgery. Additionally, mice were depleted of CD4 and/or CD8 T lymphocytes during vaccination to assess their involvement in vaccine induced anti-tumor immunity. Flow cytometry was performed to characterise changes in the proportion and activation status of immune cells associated with anti-tumor immunity.

**Results:**

Neoadjuvant vaccination combined with debulking surgery resulted in decreased tumor burden, increased survival and generation of tumor-specific immunity compared to surgery alone. Depletion of CD8 T cells completely abrogated any vaccine induced anti-tumor immune response. Conversely, CD4 depletion enhanced CD8 T cell activation resulting in complete tumor regression in 70% of mice treated with combined surgery and vaccination therapy. Tumor free survival was associated with established immunological memory as defined by the induction of effector memory T cells and resistance to rechallenge with parental AB1 mesothelioma cells.

**Conclusions:**

Neoadjuvant anti-cancer vaccination combined with partial debulking surgery induced CD8-dependent anti-tumor immunity that significantly delayed tumor outgrowth relative to surgery alone. Complete tumor eradication was observed when vaccination and surgery were performed in CD4 T cell depleted animals. This demonstrates that adjuvant immunotherapy can improve post-surgical survival following cancer debulking surgery and provides a scientific rational for clinical trials of such an approach.

## Background

Surgery is a widely used therapy for many common solid tumors, including thoracic malignancies such as non-small cell lung cancer and in some patients with malignant mesothelioma [1,2]. However, surgery alone is often not curative, as these cancers commonly relapse due to local recurrence of unresectable tumor or growth of distant metastases. While adjuvant chemotherapy or radiotherapy is often used post-surgery to eradicate sub-clinical distant metastases or residual local disease, these therapies result in limited survival benefits [3–6]; highlighting the need for the development of better adjuvant therapies.

In the last decade there has been considerable improvement in our understanding of the interaction between the host immune system and developing tumor. This has encouraged many researchers to revisit the use of adjuvant immunotherapies to target residual tumor mass. We have previously demonstrated delayed tumor development after combining debulking surgery with antitumor immunotherapy [7–9] and successfully used autologous tumor lysate as an effective anti-cancer vaccine [10,11]. Vaccination against known tumor antigens also represents an appealing therapeutic option for the eradication of residual tumor following debulking surgery with different tumor vaccine protocols showing promise in a variety of preclinical models [12–15] and cancer vaccines continue to be clinically evaluated for a range of solid cancers (reviewed in [16–18]).

However, understanding when to vaccinate in relation to surgery may have a significant impact on the outcome of the treatment. In a murine model of melanoma, neoadjuvant vaccination (i.e. prior to surgery) was shown to provide superior protection against post-surgical tumour relapse compared to adjuvant vaccination [13]. The authors assessed the presence and frequency of the key cell types associated with effective anti-tumor immunity (namely CD8 and CD4 T lymphocytes) and found that neoadjuvant vaccination enables surgery to coincide with the peak of the vaccine induced immune response, resulting in increased frequency tumor-specific CD8 T cells present in the tumor, lymph nodes and resection area. In this study we have chosen murine mesothelioma as the model because the tumors mimic their human counterparts molecularly, biologically and clinically and their immunological engagement with the host before and after surgery has been well defined [7–9]. Importantly, they appear to be sensitive to immunotherapy and, as some patients undergo debulking surgery, an ideal situation exists for possible translation to clinical studies.

## Methods

### Reagents and mice

All reagents were purchased from Sigma Aldrich (Castle Hill, NSW. Australia) unless stated otherwise. Female BALB/C (H-2K^d^) aged between 6 and 8 weeks were obtained from the Animal Resources Centre (Murdoch, Western Australia). All mice were maintained under standard specific pathogen free (SPF) housing conditions and all animal experiments were carried out according to protocols approved by the University of Western Australia Animal Ethics Committee.

### Mouse model: tumor inoculation and surgical resection of tumors

Mice were inoculated subcutaneously (s.c.) with 5×10^5^ AB1-HA cells in a total volume of 100 μl PBS on the right hand flank unless otherwise stated. For all experiments mice were randomised into groups of 5 once tumors were established (approximately 50–70 mm^2^). Mice were culled when tumors reached the maximum allowable size of 100 mm^2^ as per UWA AEC approvals. All surgical resections were performed under general anaesthetic using inhalant isoflurane. As a control for the surgical process sham surgery was performed by making incisions into the tumor mass and re-suturing, without debulking. Debulking surgery was performed via elliptic incisions, centred over the s.c. tumors. Skin flaps were elevated to expose adherent tumors. Once tumors were dissected clear of adjacent fascia, 75% of the tumor mass was removed, with preservation of the tumor pedicles to ensure blood supply (as previously described [19]). Wounds were closed using 4–0 vicryl (polyglactin 910, Ethicon, Australia) continuous sutures. Mice received 0.1 mg/kg buprenorphine intraperitoneally (i.p.) in the recovery phase for postoperative analgesia as required.

### Cell lines & vaccines

The murine mesothelioma cell line (AB1) was generated by injecting crocidolite asbestos i.p. into BALB/C mice as previously described [20] and subsequently transfected with the PR8 influenza virus haemagglutinin (HA) gene to produce AB1-HA [21].

Mouse adapted Influenza virus (PR/8/34/H1N1) was a kind gift from Dr Peter Henry (UWA School of Pharmacology, Perth Western Australia). PR8 virus was propagated in the allantoic fluid of 9-day old embryonated chicken eggs (Altona Hatchery, Forrestfield, Australia) at 37°C for 3 days and harvested as previously described [22], stored in single use aliquots at −80°C and diluted 1:100 to 1:400 in sterile PBS prior to intranasal vaccination of mice.

Generation of recombinant Modified Vaccinia Ankara expressing Influenza HA antigen. PR8 Influenza HA from the AB1-HA cell line was cloned via RT-PCR into pCR4 cloning vector using the pZeroBlunt-TOPO cloning kit. PCR primers were designed to introduce a *Pme* I restriction site and add a 6HIS-3xSTOP-*Asc* I sequence to the 5′ and 3′ end of the HA cds respectively. The *Pme* I/*Asc* I flanked HA-6HIS insert from pCR4-HA was then cloned into the modified Vaccinia Ankara (MVA) shuttle vector pZWIGR3 [23] to produce pZWIGR3-HA. To generate recombinant MVA (rMVA) expressing HA, pZWIGR3-HA was transfected into wild type MVA (wtMVA) infected BHK-21 cells and rMVA-HA positive cells purified via sequential rounds of plaque purification based on expression of the fluorescent reporter Venus. The purity of rMVA-HA stocks was confirmed by the absence of wtMVA and presence of HA via PCR prior to expansion and ultra-purification of rMVA-HA stocks for experimental use. All rMVA-HA vaccinations were via i.p. injection in a total volume of 100 μl PBS containing 5×10^5^ plaque forming units of virus.

### Flow cytometry and antibodies

Flow cytometry was performed using a BD Canto II. All antibodies are anti-mouse unless otherwise stated. Flow cytometry: αCD3-PE-Cy7 (clone 145-2C11), αCD4-PerCP-Cy5.5 (clone GK1.5), αCD8-FITC (clone 53–6.7) and αCD278(ICOS)-APC (clone C398.4A) all BioLegend, USA; αCD8-APC-ef780 (clone 53–6.7), αFoxP3-FITC (clone FJK-16 s), αIFNγ-APC (clone XMG1.2), αCD44-PerCP-Cy5.5 (clone IM7), αCCR7-PE (clone 4B12) and αCD62L-APC (Clone MEL-14) all eBioscience, USA; αKi67-PE (clone B56) (BD Biosciences, USA) and HA-Dextramer-APC (Immudex USA, LLC. Virginia USA). For depletion experiments, purified αCD4 (GK1.5) and αCD8 (YTS.169) antibodies were obtained from Absolutions Pty Ltd (Western Australian Institute for Medical Research, Perth, Western Australia). Antibodies were administered by intravenous (i.v.) injection at a dose of 150 μg per mouse on day 1 and then 100 μg i.p. per mouse every third day as indicated. T cell subset depletion was confirmed by flow cytometry on peripheral blood samples.

### Statistical analysis

Student’s *t* test was used to measure significance between two individual groups, Log rank analysis was performed on survival curves. All analysis was performed using Graph Pad Prism Software (Graph Pad Software Inc., CA, USA) and a p value <0.05 considered significant.

## Results

### Neoadjuvant vaccination delays tumor growth following debulking surgery

We have previously demonstrated that partial, but not complete debulking surgery promotes protective anti-tumour immunity when combined with adjuvant immunotherapy [7]. This is despite complete resection preventing any relapse of residual tumor growth [19]. We have continued to refine our model of tumor debulking by investigating the effect of removing different amounts of tumor on the rate of residual tumor outgrowth and overall survival (Khong et al., manuscript in preparation) When 75% of the tumor was debulked we observed that the outgrowth of the residual tumor was relatively slow, while in contrast, removing 50% or less of the tumor had no effect on tumor outgrowth. We therefore chose a 75% debulk model for this study as it better represents the many clinical scenarios where debulking surgery is the realistic goral rather than complete resection.

**Figure 1:**
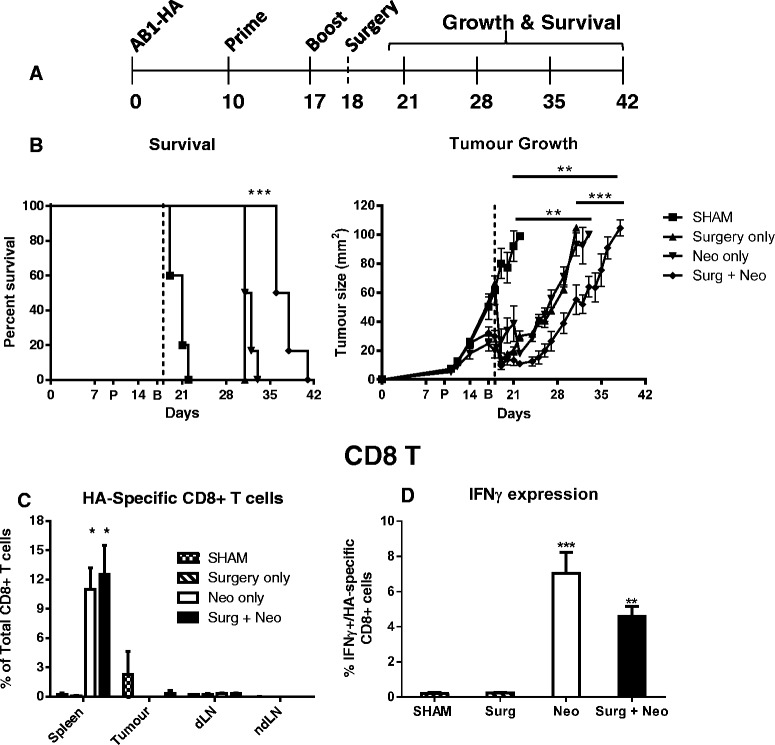
**Neoadjuvant P/B vaccination enhances delay in tumor growth following debulking surgery. (A)** Schematic of experiment design. BALB/c mice bearing AB1-HA tumor received PR8 prime vaccine (day 10) and rMVA-HA boost vaccine (day 17) alone, in combination with surgery (day 18) and tumor growth and overall survival compared to surgery only or sham surgery control groups. **(B)** Survival and growth curves showing significant survival benefit with combined therapy. **(C-D)** An increase in the proportion **(C)** and function **(D)** of HA-specific CD8 T cells was only observed in the spleens of vaccinated mice on day 21. **p < 0.01, ***p < 0.001. dLN = draining lymph node. ndLN = non-draining lymph node. Data = mean + SEM.

To determine whether the addition of PR8 prime and rMVA-HA boost (P/B) vaccination directed against the HA-neo tumour antigen (as describe above) could improve the survival benefit associated with debulking surgery, groups of AB1-HA tumor bearing mice were treated with either sham surgery (tumor incised, but not debulked), 75% debulking surgery, prime boost vaccination or a combination of neoadjuvant vaccination and surgery (Figure 1A). A significant delay in tumor growth (p < 0.001) was observed for surgery combined with vaccination group relative to other treatment groups. (Figure 1B). Groups receiving vaccination (alone or in combination with surgery) had noticeably smaller tumors on the day of surgery and a significantly higher proportion of interferon gamma (IFN-γ) expressing tumor-specific CD8 T cells when compared to non-vaccinated groups one week after vaccination (Figure 1D). However, despite all vaccinated groups demonstrating the presence of a functional tumour-specific CD8 T cell immune response, only the combination of neoadjuvant vaccination and 75% debulking surgery was associated with significantly delayed tumor growth and increased significant survival benefit (p < 0.001) relative to surgery or vaccination alone (Figure 1B).

### CD4 T cell depletion during vaccination results in significant survival benefit following debulking surgery

**Figure 2:**
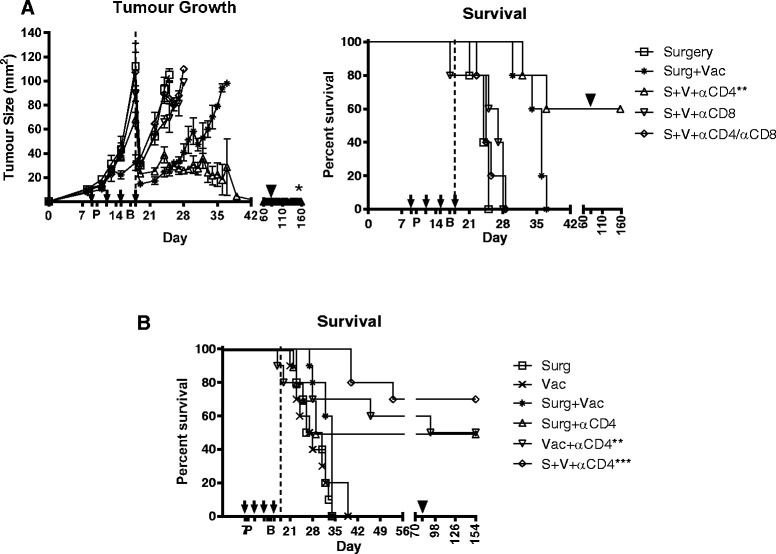
**Depletion of CD4 T cells during neoadjuvant vaccination significantly improves survival outcome after debulking surgery.** All groups of AB1-HA bearing BALB/c mice (n = 10) received 75% debulking surgery on day 18 (dotted line). Vaccinated mice received rMVA-HA i.p. 7 days apart (P/B) and depleting antibodies (black arrows) were given every 3 days, starting 1 day prior to the first vaccination (q3dx4). Surviving mice were rechallenged on day 81 (black triangle). **(A)** Tumor growth and survival data showing delay in tumor growth after combined therapy compared to surgery only. Complete tumor regression was only observed when combined therapy was performed in the absence of CD4 T cells (open triangle). **(B)** Data from repeat experiments showing overall survival following surgery or vaccination as individual or combined therapies, with or without CD4 T cell depletion. Depletion of CD4 T cells significantly improved the survival outcome relative to non-depleted controls. All surviving mice resisted tumor rechallenge. **p < 0.01, ***p < 0.001. All significant Logrank comparison are to respective untreated controls.

We hypothesised that the delay in tumor growth in vaccinated mice resulted from a vaccine induced anti-tumor immune response. To test this, we depleted two known key anti-tumour immune effector cell types, CD4 and CD8 T lymphocytes, during vaccination and compared the overall survival of each group (Figure 2A). Consistent with our earlier experiments, we observed a delay in tumor growth that was associated with a significant survival benefit (p < 0.01) when neoadjuvant vaccination was combined with 75% debulking surgery compared to surgery alone; although this was not sufficient to prevent tumor outgrowth. Depletion of CD8 T cells, either alone or in combination with CD4 T cells, completely abrogated any vaccine induced survival benefit, reducing the survival of CD8-depleted groups to that of the surgery only group (Figure 2A), indicating that CD8 T cells are essential for an effective anti-tumor immune response. Similarly, CD4 depletion during vaccination prevented any vaccine induce delay in tumor growth prior to surgery. However, in contrast to the combined surgery and vaccine group, in which all mice eventually succumb to tumor, we observed complete tumor eradication in 60% of CD4 depleted mice following debulking surgery (Figure 2A). Further experiments confirmed that CD4 depletion either prior to surgery, or during vaccination was sufficient to eradicate tumor in 50% (5/10) of treated animals and this could be increased to 70% (7/10) when vaccination was combined with surgery (Figure 2B), demonstrating that vaccination with CD4 depletion significantly enhances survival compared to surgery alone. In both sets of experiments all mice survived tumor-free for longer than 60 days post-surgery. In addition, they resisted rechallenge with the parental tumor cell-line that does not express HA (black arrow, Figure 2A & B), indicating that the vaccine-induced immune response was against shared tumor antigens on the AB1 mesothelioma cells and not solely against the transfected HA antigen.

### CD4 T cell depletion enhances CD8 T cell activation and establishes immunological protection

**Figure 3:**
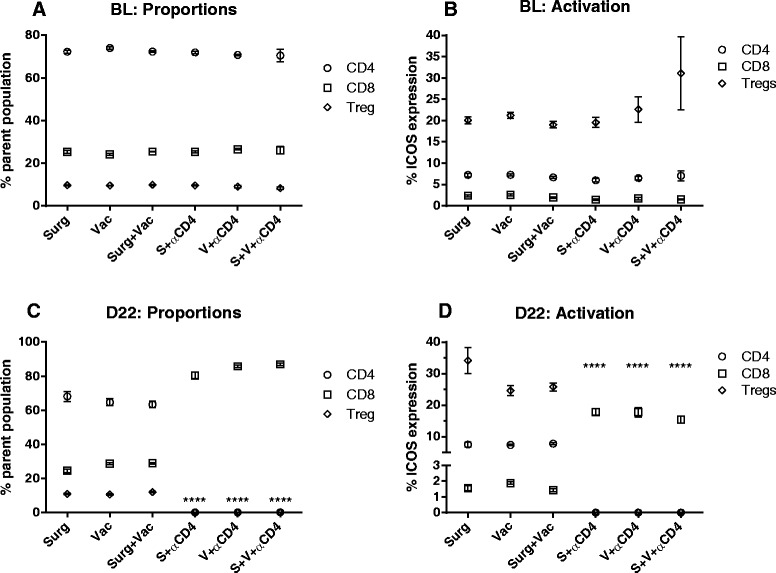
**Enhanced CD8 T cell activation following CD4 Depletion.** Flow cytometry analysis of pre (baseline) and post-treatment (day 22) peripheral blood lymphocytes taken from the same mice shown in Figure 2B. **(A-B)** The relative proportion and activation (ICOS) status of CD4, CD8 and Treg (CD4 + FoxP3+) lymphocyte subsets were similar between all groups prior to treatment. **(C-D)** CD4 T cell depletion resulted in a significant increase (****p < 0.0001) in the relative proportion and activation status of CD8 T cells (black squares) compared to the respective non-CD4 depleted groups.

**Figure 4:**
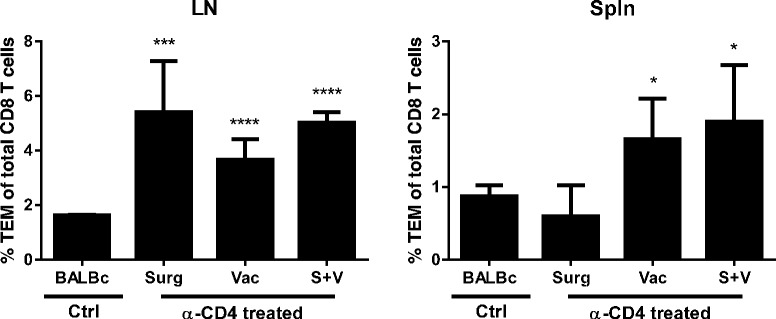
**Enhanced immunological memory following CD4 T cell depletion.** Polychromatic flow cytometry was used to determine the proportion of CD8+ CD44+ CD62L- effector memory (T_EM_) T cells in spleen (Spln) and lymph nodes (LN) of the tumor rechallenged mice from Figure 2B. Significantly more T_EM_ were observed in LN and Spln from all CD4 depleted groups compared to control mice. * = p <0.05, ***p < 0.001, ****p < 0.0001.

Having demonstrated that CD8 T cells were essential for effective anti-tumor immunity, we next assessed the immunological effect of CD4 depletion on CD8 T cells. Peripheral blood taken from mice during CD4 T cell depletion (days 7, 10, 15 and 22, shown in Figure 2B) was analysed by polychromatic flow cytometry to assess the proportion and activation status (ICOS expression) of T cell subsets. The proportion of CD4 and CD8 (as a percentage of total CD3+ lymphocytes) and FoxP3 + CD4+ regulatory T cells (Treg, as a proportion of total CD4+ T cells) was as expected, the same in all groups at baseline (day 7) and remained unchanged throughout the experiment in all non-CD4 depleted groups (Figure 3). In contrast, the proportion of CD4+ T cells and Treg were significantly depleted upon anti-CD4 mAb treatment (p < 0.0001), correlating with a significant increase in the proportion and activation status of CD8 T cells (Figure 3C-D, p < 0.0001). Sustained CD8 T cell activation was observed in CD4 depleted groups, coinciding with tumor regression (days 22–35) and ultimately long term survival (Figure 2B). CD4 T cell depletion was transient and had returned to baseline levels by day 81 when surviving mice were rechallenged with parental tumour (data not shown). Again, all surviving mice resisted rechallenge and remained tumor free until completion of the experiment (day 154). Establishment of immunological memory was consistent with the finding of a significant increase in CD44+ CD62L- effector memory T cells (T_EM_) in the spleen and lymph nodes of CD4 depleted groups at day 140 relative to naïve bearing controls (Figure 4).

## Discussion

Although surgery remains the most effective treatment option for many solid malignancies, regrowth of residual/non-resectable tumor or growth of distal metastases means that surgery is not always curative, highlighting the need to develop improved, combinatorial treatment strategies. Over the past decade our knowledge of how the immune system recognises and interacts with developing tumor has greatly expanded (reviewed in [2,24,25]), driving a renewed interest in utilising adjuvant immunotherapies in the treatment of solid tumors. One key question is whether new immunotherapies can add value to tumor debulking surgery.

In this study we assessed the impact of neoadjuvant vaccination in combination with partial debulking surgery. We chose a murine mesothelioma tumour model because it mimics its human counterpart and because it represents an ideal tumor for clinical translation. We found that vaccination induced a significant increase in CD8+ cytotoxic T lymphocytes (CTL), a key type of effector cell in anti-tumor immune responses [25]. While surgery or vaccination alone delayed tumor growth relative to controls, a significant survival benefit was only observed when surgery and vaccination were combined. However, unlike Grinshtein *et al.,* who showed that neoadjuvant vaccination protected against tumor regrowth after complete surgical resection [13], we observed that combined treatment could not prevent tumor outgrowth in our model. Instead, complete tumor eradication was only observed when combined vaccination and surgery was performed in the absence of CD4 T cells. While differences between models and vaccination protocols make it hard to directly compare our results, it is known that mesotheliomas secrete immunosuppressive cytokines such as TGF-β that may limit vaccine induced anti-tumour immunity in the tumor environment [26,27].

The requirement for CD4 T cell depletion suggests a role for regulatory T cells in limiting vaccine induced anti-tumour immunity. Treg are a subset of CD4+ T cells and are recognised as important negative regulators of immune responses [28]. Treg have been shown to promote tumor growth by limiting the efficacy of tumor-specific CD8 T cell responses and their presence within the tumour infiltrate has been correlated with poor survival prognosis for a number of different malignancies (reviewed in [29]). Therefore, consistent with our findings, vaccination protocols that involve Treg depletion or inhibition of their immunosuppressive function warrant further investigation.

It is important to consider how CD4 depletion might be achieved in a clinical setting. To date cyclophosphamide has been extensively studied for its ability to enhance anti-tumour immunity, including anti-tumour vaccination, by reducing Treg when used at low doses [30–34]. Conversely, high dose cyclophosphamide is lymphodepletive [35,36] and while the use of high dose cyclophosphamide in combination with total body irradiation is critical to increase the effectiveness of adoptive cell therapy (ACT) treatment of melanoma patients [37,38], it is not without associated toxicity and the requirement of CD4 depletion during ACT has been questioned [39].

Recent studies have also shown that targeting the surface receptors cytotoxic T-lymphocyte antigen 4 (CTLA-4) and programmed death-1 (PD-1) on both effector and regulatory T cells, a process known as checkpoint blockade, can dramatically improve anti-tumor immunity [40–44], especially in advanced stage melanoma [45–47]. These new advances in immunotherapy suggest a role for adjuvant immunotherapy to be included in anti-cancer vaccination protocols, particularly when combined with conventional treatments such as debulking surgery.

Taken together, this study demonstrates that neoadjuvant anti-tumor vaccination combined with partial debulking surgery is capable of inducing effective CD8 T cell dependent anti-tumor immunity. Furthermore, depletion of CD4 T cells during vaccination produced complete tumor eradication and established immunological memory that could protect against subsequent tumor growth.

## Conclusions

These results suggest that prior to performing debulking surgery, administration of an immunotherapy consisting of transient CD4 T cell, or more specifically Treg depletion combined with anti-tumor vaccination is an effective therapeutic strategy to enhance survival. These results provide a logical basis for development of adjuvant immunotherapies for patients undergoing cancer surgery in whom complete tumor resection is not likely to be achieved, or who may achieve complete resection, but at high risk or relapse.

## Abbreviations

AB1-HA: Murine mesothelioma cell line
ACT: Adoptive cell transfer
BALB/C: Albino mouse
CTL: Cytotoxic T lymphocyte
CTLA-4: Cytotoxic T lymphocyte antigen 4
FoxP3: Forkhead box transcription factor 3
HA: Haemagglutinin
ICOS: Inducible T-cell COStimulator
IFN-γ: Interferon gamma
i.p.: Intraperitoneal
mAb: Monoclonal antibody
MVA: Modified Vaccinia Ankara
PBS: Phosphate buffered saline
P/B: Prime Boost vaccination
PD-1: Programmed cell death protein 1
PR8: Influenza A PR/8/34 H1N1 Mt Saini strain
s.c.: Subcutaneous
T_EM_: T effector memory
TGF-β: Tumour growth factor beta
Treg: Regulatory T cell
